# Supplementary material for: Microwave Transmittance Technique Using Microstrip Patch Antennas, as a Non-Invasive Tool to Determine Soil Moisture in Rhizoboxes
Source: Sensors (Basel). 2020 Feb 20;20(4):1166. doi: 10.3390/s20041166 (PMC7070758; doi:10.3390/s20041166)
Supplement: Supplementary file 1 [file sensors-20-01166-s001.pdf]

Supplementary Material

In Table 1 of the article, the calculation of the dielectric properties of the test liquids at 25 ° C and f = 4.8 GHz was presented.

Figure S1A–D are showing the dependences of S_{21} on different parameters, as (A) tanδ X S_{21} (dB)¸ (B) $|\varepsilon^*|$ X S_{21} (dB); (C) ε' X S_{21} (dB) and (D) ε'' X S_{21} (dB).

In Figure 7 of the paper the selected parameter is presented, which was considered the most suitable for this type of measurements.

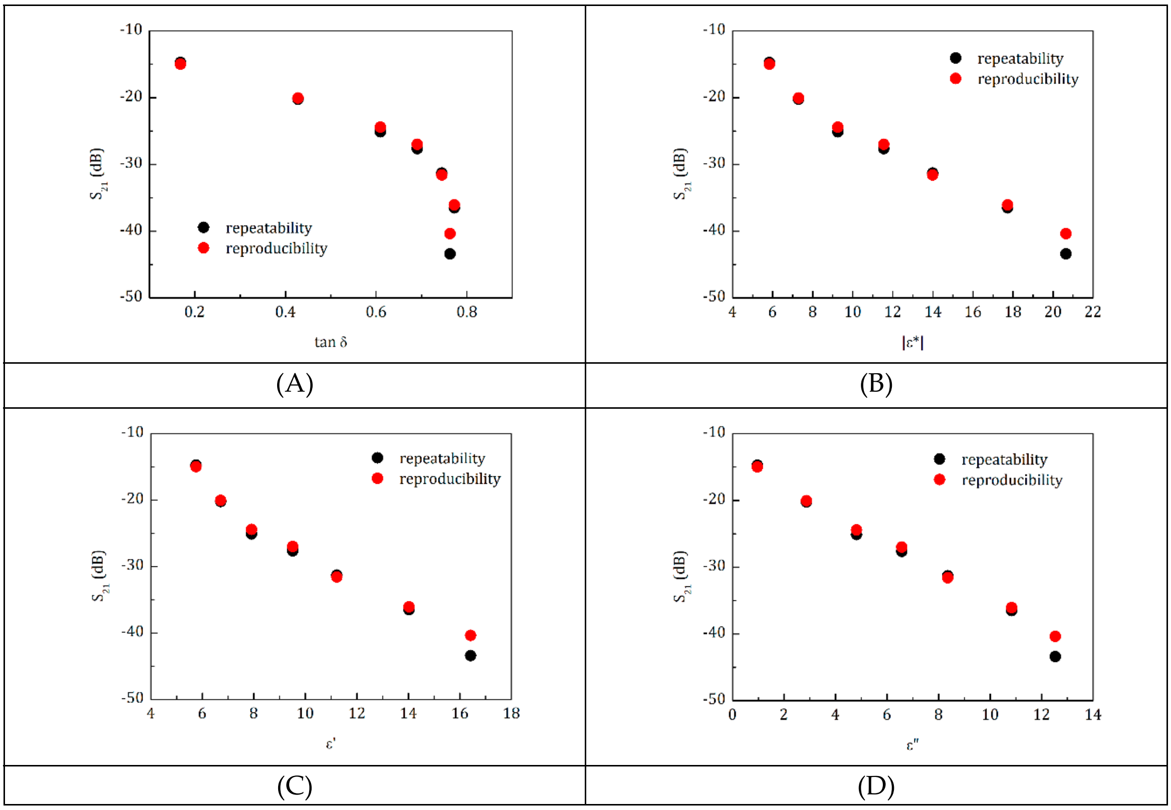

Figure S1. The graphic that showing the dependences of S_{21} on different parameters, as A) tanδ X S_{21} (dB)¸ (B) $|\varepsilon^*|$ X S_{21} (dB); (C) ε' X S_{21} (dB) and (D) ε'' X S_{21} (dB).
